# Optimization of Chromium Removal by the Indigenous Bacterium *Bacillus* spp. REP02 Using the Response Surface Methodology

**DOI:** 10.5402/2011/951694

**Published:** 2011-10-19

**Authors:** C. K. Venil, V. Mohan, P. Lakshmanaperumalsamy, M. B. Yerima

**Affiliations:** ^1^Division of Food Microbiology and Biotechnology, Department of Food Science and Technology, Pondicherry University, Pondicherry 605 014, India; ^2^Forest Pathology Laboratory, Forest Protection Division, Institute of Forest Genetics and Tree Breeding, Coimbatore 641 002, Tamil Nadu, India; ^3^Karpagam University, Coimbatore 641 021, Tamil Nadu, India; ^4^Department of Microbiology, Faculty of Sciences, Usmanu Danfodiyo University Sokoto, Sokoto, Nigeria

## Abstract

An indigenous bacterium, *Bacillus* REP02, was isolated from locally sourced chromium electroplating industrial effluents. Response surface methodology was employed to optimize the five critical medium parameters responsible for higher % Cr^2+^ removal by the bacterium *Bacillus* REP02. A three-level Box-Behnken factorial design was used to optimize K_2_HPO_4_, yeast extract, MgSO4, NH_4_NO_3_, and dextrose for Cr^2+^ removal. A coefficient of determination (*R* ^2^) value (0.93), model *F*-value (3.92) and its low *P*-value (*F* < 0.0008) along with lower value of coefficient of variation (5.39) indicated the fitness of response surface quadratic model during the present study. At optimum parameters of K_2_HPO_4_ (0.6 g L^−1^), yeast extract (5.5 g L^−1^), MgSO_4_ (0.04 g L^−1^), NH_4_NO_3_ (0.20 g L^−1^), and dextrose (12.50 g L^−1^), the model predicted 98.86% Cr^2+^ removal, and experimentally, 99.08% Cr^2+^ removal was found.

## 1. Introduction

Widespread industrial applications of chromium and the resultant effluent discharge affect the environment adversely [1]. Conventional chemical, physical methods (chemical precipitation, chemical oxidation or reduction, ion exchange, filtration, electrochemical treatment, reverse osmosis, membrane technologies, evaporation recovery, etc.), and activated sludge biological treatment for removal of chromium are generally efficient in removing the bulk of metal from solution at high or moderate concentrations, whereas they may be ineffective or extremely expensive especially when the metals in solution are at low concentration [2]. As a consequence, their limits (high cost, high reagent requirements, etc.) become more pronounced when voluminous effluents containing complexing organic matter and low metal contamination were to be treated. Compared with these conventional methods, biological treatment shows some advantages, such as low operation cost, steady effect, easy recovery of some valuable metals [3]; incidentally biotechnological approaches with right designs can succeed in treating such niches [4]. 

A wide variety of microorganisms such as bacteria, yeast, algae, protozoa, and fungi found in waters receiving industrial effluents, have developed the capabilities to protect themselves from heavy metal toxicity by various mechanisms such as adsorption, uptake, methylation, oxidation, and reduction. Many microorganisms have been reported to hold inbuilt competence to reduce the highly soluble and toxic Cr^6+^ to the less soluble and less toxic Cr^3+^,  for example*, Acinetobacter *and *Ochrobactrum *[5], *Arthrobacter *[6], *Pseudomonas *sp. [7], *Serratia marcescens *[8], *Ochrobactrum *sp. [9], *Bacillus *sp. [10], *Desulfovibrio vulgaris *[11], and *Cellulomonas *spp. [12].

Among microorganisms, bacteria are better candidates for heavy metal removal as these are easy to culture, easy to handle and have very simple nutritional requirements. Several studies on metal removal by different bacterial species have been carried out by optimizing the medium parameters applying one variable at a time or response surface methodology (RSM). One variable at a time method is laborious, time consuming to perform experiments, not possible to obtain accurate optimum conditions and to detect the frequent interactions occurring between two or more factors [13, 14]. On the other hand, RSM is a combination of mathematical and statistical techniques used for developing, improving, and optimizing the processes. It is used to evaluate the relative significance of several affecting factors even in the presence of complex interactions. 

Although heavy metal removal efficiency of the microorganisms is a confirmed fact, there is possibility for existence of new microorganisms in local effluents because of the influence of the local socioeconomic factors over the heavy metal effluents of the area. The present study was aimed to isolate a potent indigenous bacterium from such locally sourced effluents from chromium electroplating industries and study its efficiency in chromium uptake under various interactive parameters by applying RSM.

## 2. Materials and Methods

### 2.1. Sampling

Effluent samples were collected locally in screw capped sterilized bottles from Roots Industries Pvt. Ltd., Kurudampalayam, Coimbatore 641017, Tamil Nadu, India that uses chromium for metal plating.

### 2.2. Isolation of Chromium Resistant Bacteria

For isolation of chromium-resistant bacteria, 100 *μ*L of the effluent sample was spread on Luria-Bertani (LB) agar plates. The medium was autoclaved at 121°C and 15 lbs for 15 min. The growth of the bacterial colonies was observed after 24 h of incubation at 37°C. Effect of Cr^6+^  on the growth of bacterial isolates was determined in a minimal medium which contained (g/L): NH_4_Cl, 1.0; CaCl_2_·H2O, 0.001; MgSO_4_·7H_2_O, 0.2; FeSO_4_·7H_2_O, 0.001; sodium acetate, 5.0; yeast extract, 0.5; K_2_HPO_4_, 0.5 (pH 7) supplemented with K_2_Cr_2_O_7_ [15]. It was again incubated at 37°C for 24 h. This process was repeated with successively higher concentrations of Cr^6+^, until the minimum inhibitory concentration (MIC) of bacterial isolate was obtained, and the isolate was identified as *Bacillus *spp. REP02.

### 2.3. Chromium Assay

Cr^6+^  concentrations was determined by 1–5 diphenylcarbazide method (EPA, 2006) using UV—Vis spectrophotometer [16] at 540 nm. The initial and the final concentration of chromium used in batch mode studies were calculated by estimating the concentration of chromium spectrophotometrically. From the difference in concentration, the removal efficiencies of the bacteria was calculated.

### 2.4. Experimental Design

The Box-Behnken factorial design was used to optimize the Cr^2+^ removal efficiency of *Bacillus *sp. REP02. This experimental design consisting of three levels (low, medium and high coded as −1, 0, and +1) in 46 runs were performed in duplicate to optimize the levels of five chosen key medium parameters, that is, K_2_HPO_4_, yeast extract, MgSO_4_, NH_4_NO_3_, and dextrose. For statistical calculations the five independent variables were designated as *X*_1_, *X*_2_, *X*_3_, *X*_4_, and *X*_5_ , respectively, and were coded according

(1) $$X_{i}=\frac{X_{i}-X_{0}}{\Delta X_{i}},$$

where *X*_*i*_ is the real value of an independent variable, *X*_0_ is the real value of an independent variable at the centre point and Δ*X*_*i*_ is the step change value [17]. The lowest and highest levels of the variables were K_2_HPO_4_ (0.20 to 1.0 g L^−1^), yeast extract (1 to 10 g L^−1^), MgSO_4_ (0.04 to 0.4 g L^−1^), NH_4_NO_3_ (0.2 to 1.0 g L^−1^), and dextrose (5 to 20 g L^−1^). The range of variables selected (Table 1) was based on preliminary experiments in which broad ranges were used and the range for each variable selected during the present study was the one in which metal removal was maximum. The metal removal efficiency of *Bacillus *sp. REP02 was multiply regressed with respect to the different parameters by the least square methods as follows:

(2) $$Y=\beta_{0}+\overset{}{\underset{}{\sum }}\beta_{i}x_{i}+\overset{}{\underset{}{\sum }}\beta_{ii}x_{i}^{2}+\overset{}{\underset{}{\sum }}\beta_{ij}x_{i}x_{j},$$

where *Y* is the predicted response variable, *β*_0_, *β*_*i*_, *β*_*ii*_, and *β*_*ij*_ are constant regression coefficients of the model, *x*_*i*_ and *x*_*j*_ (*i* = 1,3; *j* = 1,3, *i* 6.25*j*) represent the independent variables in the form of coded values. The accuracy and fitness of the above model was evaluated by coefficient of determination (*R* ^2^) and *F* value. The predicted values for Cr^2+^ removal were obtained by applying quadratic model (Design Expert software version 7.1.5, Stat Ease). The optimum values of the variable parameters for metal removal were obtained by solving the regression equation, analyzing the contour plots and constraints for the variable parameters using the same software. 

### 2.5. Validation Experiment

The mathematical model generated during RSM performance was validated by conducting experiments on given optimal medium setting.

## 3. Results and Discussion

Microbes can develop a high resistance to heavy metals by a variety of mechanisms to remove ions, such as adsorption to cell surfaces, complexation by exopolysaccharides, intracellular accumulation or precipitation [18, 19]. That's why isolating microbes from polluted environments would represent an appropriate practice to select metal-resistant strains that could be used for heavy metal removal and bioremediation purposes [4, 20]. The present study used a similar approach and isolated bacteria accordingly from the electroplating effluents.

Among the bacteria isolated from the electroplating effluents and selected for testing, *Bacillus *spp. REP02 was the only bacterium able to grow *in vitro* into the electroplating effluents, demonstrating a real potential to adaptation to this polluted environment. This characteristic made it potentially useful for both chromium uptake and biosorption in the inactivate state, which are the two main strategies for the bioremediation of effluents polluted by heavy metals. 

Many sea weeds, bacteria, yeasts, and filamentous fungi have already been investigated for metal-binding capacities and bacteria seem among the most promising, since their cell wall surface contains many functional groups of carboxyl, hydroxyl, sulphydryl, amino groups and phosphate group of lipids, proteins and polysaccharides having ability to bind metal ions [21–23]. Nevertheless, most of these studies have been performed using synthetic metal solutions. On the other hand, this research using a bonafide electroplating effluent demonstrated the potential of *Bacillus *spp. REP02 to remove the chromium from electroplating effluent. 

In this study, the combination of the five parameters investigated under batch studies, that is, K_2_HPO_4_, yeast extract, MgSO_4_, NH_4_NO_3_, and dextrose demonstrated maximum % removal of chromium by *Bacillus *spp. REP02. Interactive effect of these five parameters confirmed their chromium removal efficiency. The results of 46 run Box-Behnken design for five medium parameters chosen for optimization of chromium removal are shown in Table 2. It shows the % removal efficiency of *Bacillus *spp. REP02 ranging from 34.97% to 98.86% corresponding to the combined effect of the five parameters in their specific ranges. The experimental results suggest that these parameters strongly support the chromium removal by the isolate *Bacillus *spp. REP02. Lowest chromium removal efficiency of 34.97% was observed under the following conditions in the 4th run: K_2_HPO_4_ (0.6 g L^−1^), yeast extract (5.5 g L^−1^), MgSO_4_ (0.22 g L^−1^), NH_4_NO_3_ (1 g L^−1^), and dextrose (5 g L^−1^). Chromium removal efficiency above 98% was observed in the 25th run when the parameters were at K_2_HPO_4_ (0.6 g L^−1^), yeast extract (5.5 g L^−1^), MgSO_4_ (0.04 g L^−1^), NH_4_NO_3_ (0.2 g L^−1^), and dextrose (12.5 g L^−1^). This suggests that MgSO_4_, NH_4_NO_3_, and dextrose had a profound influence on the chromium removal efficiency while interacting with other parameters at optimum levels. 

On several occasions it has been reported that the cell wall of bacteria responds to the culture medium and other properties of the environment by greatly changing its composition and chemical-physical properties [24, 25]. Moreover, the C : N ratio in the medium can affect the amount of structural compounds and other chemical groups of the cell wall [24]. In this study, *Bacillus *spp. REP02 cultured on the medium containing dextrose as carbon source and ammonium nitrate as nitrogen source displayed a higher chromium removal than that cultured on the medium containing other sources. This result is of great significance for the application of this method in industry, since dextrose is a low-cost material; its use as a source of carbon would reduce the generally very expensive metal removal. 

 The results obtained (Table 2) from Box-Behnken design fitted to a second order polynomial equation to explain the chromium removal efficiency of the five parameters is given in

(3) $$\begin{array}{cc} Y & =+82.77-7.58A-0.50B-4.37C-6.29D+5.78E\\ & \;+3.31AB\;-0.020AC-4.95AD-2.29AE+0.15BC\\ & \;-13.20BD-0.17BE\;+7.19CD-4.00CE+13.00DE\\ & \;-12.48A^{2}-15.77B^{2}-3.50C^{2}\;-2.34D^{2}\;-14.35E^{2}, \end{array}$$

where *Y* is the predicted response (% removal), *A*, *B*, *C*, *D*, and *E* are the coded values of K_2_HPO_4_, yeast extract, MgSO_4_, NH_4_NO_3_, and dextrose, respectively.

Significance of each coefficient was determined by Student's *t*-test and *P* values. Analysis of variance (ANOVA) results of this model are presented in Table 3. The value of *R* ^2^ and adjusted *R* ^2^ is close to 1.0, which is very high and has advocated a high correlation between the observed and the predicted values. This means that regression model provides an excellent explanation of the relationship between independent variables (parameters) and the response (chromium removal).

**Figure 1:**
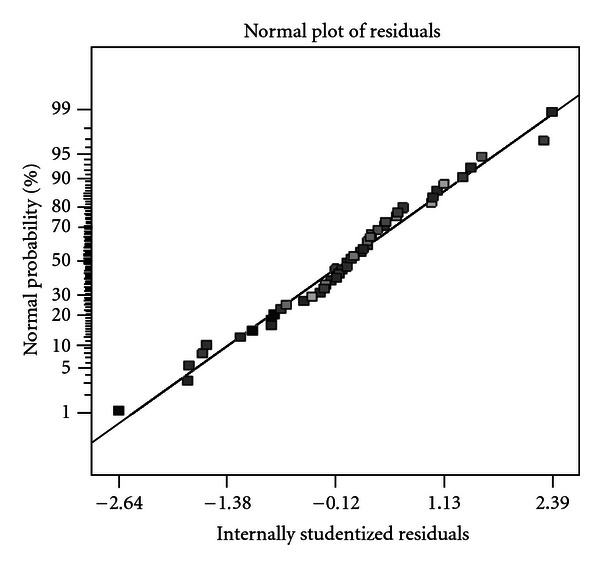
The internally studentized residuals and normal % probability plot of Cr^2+^ removal by *Bacillus* spp. REP02.

Chromium removal efficiency up to 98.86% and the remaining left over of the chromium in the effluents show how well the model satisfies the assumptions of the analysis of variance. The model adequacy check is an important part of the data analysis procedure, as the approximating model would give poor or misleading results if it were an inadequate fit. This is done by looking at the residual plots, which are examined for the approximating model [26]. The significant value (<0.05) revealed that the quadratic model is statistically significant for the response, and therefore, it can be used for further analysis. The normal probability and the internally studentized residuals plot are shown in Figure 1 for % chromium removal efficiency. The internally studentized residuals measure the number of standard deviations separating the actual and predicted values. Figure 1 shows that neither response transformation was needed nor there was any apparent problem with normality. 

Usually, it is essential to ensure that selected model is providing an adequate approximation to the real system. By applying the diagnostic plots such as the predicted versus actual value plot, the model adequacy can be judged. 

### 3.1. Effect of Interactive Parameters

The experimental design employed with five process parameters to evaluate their effect showed highest efficiency of *Bacillus *spp. REP02 in chromium removal. To indicate the interactive effect of the five parameters on chromium removal, contour plots were generated.

### 3.2. Effect of K_2_HPO_4_ and Yeast Extract

**Figure 2:**
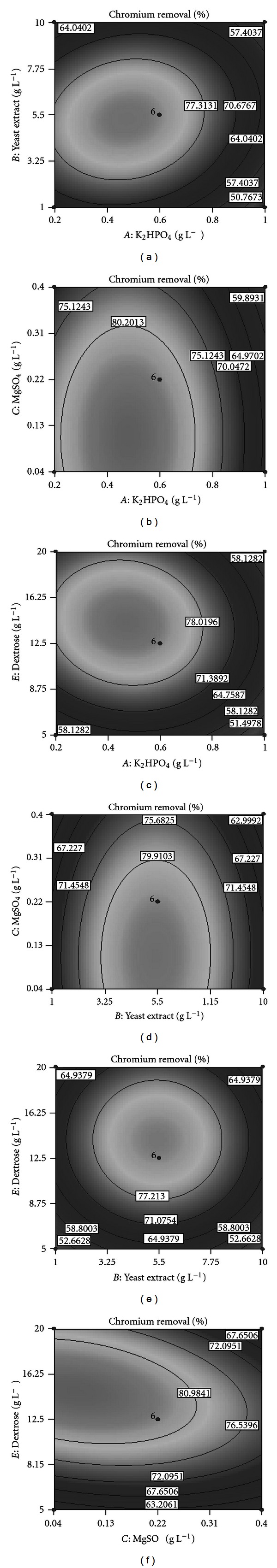
Contour surface plot for the removal of chromium by Bacillus spp. REP02—A function of K_2_HPO_4_ and yeast extract. Contour surface plot for the removal of chromium by *Bacillus* spp. REP02—A function of K_2_HPO_4_ and MgSO_4_. Contour surface plot for the removal of chromium by *Bacillus* spp. REP02—A function of K_2_HPO_4_ and dextrose. Contour surface plot for the removal of chromium by Bacillus spp. REP02—A function of yeast extract and MgSO_4_. Contour surface plot for the removal of chromium by *Bacillus* spp. REP02—A function of yeast extract and dextrose. Contour surface plot for the removal of chromium by *Bacillus* spp. REP02—A function of MgSO_4_ and dextrose.

The results of the RSM study on the combined effect of K_2_HPO_4_ and yeast extract in chromium removal are shown in the form of contour plot (Figure 2(a)). At K_2_HPO_4_ (0.75 g L^−1^) and yeast extract (6 g L^−1^), the chromium removal efficiency was 77.31% which declines to 50.76% at K_2_HPO_4_ (0.90 g L^−1^) and yeast extract (1.5 g L^−1^), respectively. The optimum values of the parameters, namely, K_2_HPO_4_ and yeast extract can be analyzed by checking the maxima formed by the *X* and *Y* coordinates of the plot. Phosphate sources play a crucial role in cellular respiration and metabolism of the microbes which induces the microbe to uptake the metal ions [27]. Yeast extract was found to influence chromium removal more than the inorganic media components. This indicates that the optimized media composition varies greatly with respect to the desired response and application. The nutrient requirement for optimum chromium removal from the effluents depends on the nature of microbial species employed.

### 3.3. Effect of K_2_HPO_4_ and MgSO_4_

The combined effects of K_2_HPO_4_ and MgSO_4_ in chromium removal are shown in the form of contour plot (Figure 2(b)). The chromium removal efficiency was 59.89% at K_2_HPO_4_ (1.0 g L^−1^) and MgSO_4_ (0.4 g L^−1^), and the % chromium removal efficiency increased to 80.20% at K_2_HPO_4_ (0.5 g L^−1^) and MgSO_4_ (0.3 g L^−1^). There are very limited data in the literature concerning the influence of microbes on the effectiveness of phosphate amendments for metal removal. *Bacillus *spp. REP02 was able to utilize Mg^2+^ ions when the concentration of this ion was low in simple medium, thereby stimulating chromium removal. Bacteria may enhance the uptake of heavy metals by increasing Mg^2+^-soluble fraction by dissolution and desorption due to the secretion of protons [28].

### 3.4. Effect of K_2_HPO_4_ and Dextrose

The results of the Box-Behnken study on the combined effect of K_2_HPO_4_ and dextrose in chromium removal are shown in the form of contour plot (Figure 2(c)). At K_2_HPO_4_ (0.95 g L^−1^) and dextrose (5.5 g L^−1^), the chromium removal efficiency was 51.49% which increased to 78.01% at K_2_HPO_4_ (0.75 g L^−1^) and dextrose (14 g L^−1^), respectively. The optimum values of the parameters namely, K_2_HPO_4_ and dextrose, can be analyzed by checking the maxima formed by the *X* and *Y* coordinates of the plot.

The efficiency of dextrose in the medium can be explained by the fact that *Bacillus *spp. REP02 is a heterotroph and is capable of utilizing dextrose as a carbon source. Most of the *Bacillus *spp. utilizes glucose as a nutrient substrate [29]. Our result clearly indicates that *Bacillus *spp. REP02 is capable of gaining energy from dextrose as an electron donor and ferric iron as an electron acceptor. Under oxygen-limiting conditions, ferric iron can be reduced to ferrous iron. The dissimulatory ferric iron reduction under anoxic state runs usually according to the following reaction stated by Coates et al. [30]

(4) $$\begin{array}{cc} & {\text{C}}_{6}{\text{H}}_{12}{\text{O}}_{6}+2{\text{H}}_{2}\text{O}+8\text{F}{\text{e}}^{3+}\\ & \;\;\overset{\text{Bacteria}}{\to }2\text{C}{\text{H}}_{3}\text{COOH}+2\text{C}{\text{O}}_{2}+8\text{F}{\text{e}}^{2+}+8{\text{H}}^{+}. \end{array}$$

### 3.5. Effect of Yeast Extract and MgSO_4_

The combined effects of yeast extract and MgSO_4_ in chromium removal are shown in the form of contour plot (Figure 2(d)). The chromium removal efficiency was 62.99% at yeast extract (1.0 g L^−1^) and MgSO_4_ (0.4 g L^−1^), and the % chromium removal efficiency increased to 79.91% at yeast extract (5.5 g L^−1^) and MgSO_4_ (0.3 g L^−1^).

It has been shown that for some microbes, high-nitrogen conditions repressed metal removal to some degree. However, nutrient limitation, especially nutrient nitrogen stimulated removal of heavy metals in most species investigated to date [31].

### 3.6. Effect of Yeast Extract and Dextrose

The interactive effect of yeast extract and dextrose in chromium removal was shown in the form of contour plot in Figure 2(e). At yeast extract (1.75 g L^−1^) and dextrose (6.0 g L^−1^), the chromium removal efficiency was 52.66% which increases to 77.21% at yeast extract (5.5 g L^−1^) and dextrose (10 g L^−1^), respectively. The optimum values of the parameters, namely, yeast extract and dextrose can be analyzed by checking the maxima formed by the *X* and *Y* coordinates of the plot.

It was observed that the propensity of *Bacillus *spp. REP02 to chromium was dramatically enhanced by the increase of yeast extract strength. The chromium ions have a strong affinity for organic materials such as yeast extract. Thus, there are two possible explanations for the toxicity decrease of chromium when increasing yeast extract strength: the organic matter reacts with chromium ions to form compounds that are less toxic than the ions themselves, and/or the ions adsorbed on the surface of particles are rendered less toxic [32].

The increase of dextrose strength influences the chromium removal by *Bacillus *spp. REP02. This is probably due to the increase of metabolic activity. It was found that the chromium transport into the bacterial cell depends on energy, therefore, it is glucose dependent [33]. Thus, at high dextrose strength, chromium removal was enhanced.

### 3.7. Effect of MgSO_4_ and Dextrose

The combined effects of MgSO_4_ and dextrose in chromium removal are shown in the form of contour plot (Figure 2(f)). The chromium removal efficiency was 63.20% at MgSO_4_  (0.22 g L^−1^) and dextrose (5.0 g L^−1^), and the % chromium removal efficiency increased to 80.98% at MgSO_4_ (0.25 g L^−1^) and dextrose (14 g L^−1^), respectively. 

The occurrence of binding chromium ions in an ion-rich medium might be explained by the binding constants of various ligands that could be responsible for the heavy metal binding. The binding constants for various functional groups such as carboxylates, sulfur groups and amino groups have an overall higher binding affinity for the various metal ions studied [34]. Because of the binding constants, heavy metal binding to the various functional groups have higher stability constants, and it stands to reason that the metals would bind before the hard cations would. This would explain the specificity of chromium binding.

### 3.8. Validation of the Model

**Figure 3:**
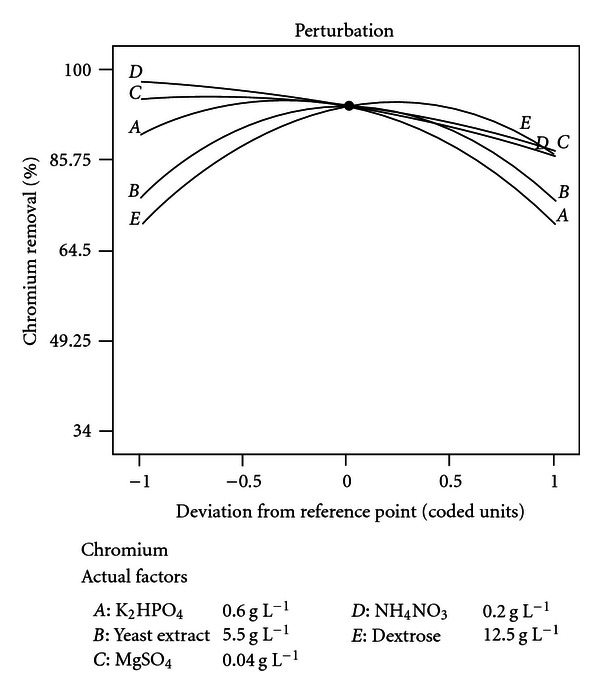
Perturbation graph showing the optimum values of the medium components.

The maximum experimental response for chromium removal was 98.86%, whereas predicted response was 99.08% indicating a strong agreement between them. The optimum values of the tested variables for maximum chromium removal by* Bacillus *spp. REP02 were K_2_HPO_4_ (0.6 g L^−1^), yeast extract (5.5 g L^−1^), MgSO_4_ (0.04 g L^−1^), NH_4_NO_3_ (0.2 g L^−1^), and dextrose (12.5 g L^−1^) as shown in perturbation graph (Figure 3). 

In an attempt to optimize industrial conditions for chromium removal, scale-up study was carried out in a jar fermentor by using medium under optimum conditions. The results are encouraging (99.05%) for optimization under pilot scale or industrial scale conditions, which could eventually reach out to actual industrial applications after due scientific compatibility study.

## 4. Concluding Remarks

In the present research, a potent indigenous bacterium *Bacillus *spp. REP02 was isolated from locally sourced electroplating effluents and its potential to uptake chromium tested by employing response surface methodology. This design helped in locating the optimum levels of the most significant medium parameters which contribute to the maximum metal removal. RSM, when employed, not only demonstrated increased metal removal efficiency of the test organism at the optimized conditions but also proved to be simple, efficient, and time and material saving. The test organism was quite capable in Cr^2+^ removal from electroplating effluents and thus has the potential to be exploited for treatment of chromium containing industrial effluents before their discharge into water bodies.

## Figures and Tables

**Table 1:** Independent variables and their levels in the experimental design.

Independent variables (g L^−1^)	Symbols	−1 Level	+1 Level
K_2_HPO_4_	A	0.2	1
Yeast extract	B	1	10
MgSO_4_	C	0.04	0.4
NH_4_NO_3_	D	0.2	1
Dextrose	E	5	20

**Table 2:** Experimental design and results of the Box-Behnken design.

Run	*A*: K_2_HPO_4_	*B*: Yeast extract	*C*: MgSO_4_	*D*: NH_4_NO_3_	*E*: Dextrose	Chromium %	
	(g L^−1^)	(g L^−1^)	(g L^−1^)	(g L^−1^)	(g L^−1^)	Experimental	Predicted
1	0.2	5.5	0.22	0.6	5	65.85	65.43
2	0.2	5.5	0.22	0.6	20	87.08	81.58
3	0.6	5.5	0.22	0.2	20	66.55	65.15
4	0.6	5.5	0.22	1	5	34.97	35.00
5	0.6	10	0.22	0.2	12.5	85.75	86.09
6	0.6	1	0.04	0.6	12.5	56.97	68.53
7	0.6	10	0.22	1	12.5	48.97	49.32
8	0.6	5.5	0.22	1	20	69.76	68.55
9	1	5.5	0.4	0.6	12.5	54.85	54.82
10	1	5.5	0.22	0.6	5	37.09	40.32
11	0.2	5.5	0.22	1	12.5	67.98	64.17
12	0.6	10	0.4	0.6	12.5	65.97	64.03
13	0.6	1	0.4	0.6	12.5	58.66	58.77
14	0.6	5.5	0.22	0.6	12.5	85.08	79.48
15	0.6	1	0.22	0.6	20	57.87	59.09
16	0.6	5.5	0.04	1	12.5	68.98	67.81
17	0.6	5.5	0.22	0.6	12.5	87.98	89.03
18	0.6	5.5	0.04	0.6	5	54.09	59.51
19	0.2	5.5	0.04	0.6	12.5	76.98	78.71
20	0.2	1	0.22	0.6	12.5	65.54	65.90
21	0.6	10	0.22	0.6	5	45.96	46.53
22	0.2	10	0.22	0.6	12.5	65.86	68.27
23	0.6	5.5	0.4	0.2	12.5	78.76	75.65
24	1	1	0.22	0.6	12.5	37.98	34.13
25	0.6	5.5	0.04	0.2	12.5	98.86	99.08
26	0.2	5.5	0.4	0.6	12.5	56.98	60.01
27	1	5.5	0.22	0.6	20	50.05	55.84
28	0.6	5.5	0.04	0.6	20	88.76	79.07
29	1	10	0.22	0.6	12.5	51.53	49.74
30	0.6	1	0.22	1	12.5	74.65	77.26
31	1	5.5	0.22	0.2	12.5	69.43	71.60
32	0.6	5.5	0.22	0.6	12.5	76.38	72.96
33	0.6	1	0.22	0.6	5	64.31	57.20
34	1	5.5	0.22	1	12.5	53.86	49.12
35	1	5.5	0.04	0.6	12.5	74.93	81.97
36	0.6	10	0.22	0.6	20	38.85	37.75
37	0.6	5.5	0.4	1	12.5	81.64	73.45
38	0.6	5.5	0.22	0.2	5	83.75	79.58
39	0.2	5.5	0.22	0.2	12.5	63.76	66.86
40	0.6	5.5	0.22	0.6	12.5	79.05	82.77
41	0.6	10	0.04	0.6	12.5	63.67	67.21
42	0.6	5.5	0.22	0.6	12.5	83.61	83.60
43	0.6	5.5	0.4	0.6	20	65.54	62.33
44	0.6	1	0.22	0.2	12.5	58.65	58.25
45	0.6	5.5	0.22	0.6	12.5	85.38	82.77
46	0.6	5.5	0.4	0.6	5	46.86	48.76

**Table 3:** Analysis of variance (ANOVA), regression coefficient estimate and test of significance for Cr^2+^ removal (response surface quadratic model).

Source	Sum of squares	df	Mean square	*F* value	*P*-value
Model	8067.895	20	403.39	3.92	0.0008
*A*-K_2_HPO_4_	918.2415	1	918.24	8.93	0.0062
*B*-Yeast extract	4.070306	1	4.07	0.04	0.8439
*C*-MgSO_4_	306.075	1	306.08	2.98	0.0969
*D*-NH_4_NO_3_	633.7806	1	633.78	6.16	0.0201
*E*-Dextrose	534.5344	1	534.53	5.20	0.0314
*AB *	43.75823	1	43.76	0.43	0.5202
*AC *	0.0016	1	0.00	0.00	0.9969
*AD *	97.91103	1	97.91	0.95	0.3386
*AE *	21.02223	1	21.02	0.20	0.6551
*BC *	0.093025	1	0.09	0.00	0.9762
*BD *	696.4321	1	696.43	6.77	0.0154
*BE *	0.112225	1	0.11	0.00	0.9739
*CD *	206.7844	1	206.78	2.01	0.1686
*CE *	63.92003	1	63.92	0.62	0.4380
*DE *	675.74	1	675.74	6.57	0.0168
*A^2^*	1360.048	1	1360.05	13.22	0.0013
*B^2^*	2170.927	1	2170.93	21.10	0.0001
*C^2^*	106.6674	1	106.67	1.04	0.3183
*D^2^*	47.96591	1	47.97	0.47	0.5010
*E^2^*	1797.403	1	1797.40	17.47	0.0003
Residual	2571.918	25	102.88		
Lack of Fit	2485.731	20	124.29	7.21	0.0188
Pure Error	86.18655	5	17.24		
Cor Total	10639.81	45			

*R^2^*: 0.93; Adj *R^2^*: 0.8649; Pred *R^2^*: 0.0538; CV: 5.3.
